# Absence of the Radius

**Published:** 1879-02

**Authors:** 


Absence of the Radius.—Dr. Julius Kaczander, of Budapest, reports the following deformity in a still-born child (male) almost at full time: The left forearm was very much shortened, the thumb of the left hand was wanting, and the hand itself fixed at right angles to the forearm.

At the lower end of the humerus, the external condyle is very prominent; there is no eminentia capitata; the trochlea is broad and very shallow. The radius is wanting. In place of the olecranon, we find a convex tubercle, and flexion of the ulna is very limited; the styloid process of the ulna is wanting. There is no scaphoid bone, no multangulum majus; the bones of the thumb are absent. The os lunatum is united with the ulna by means of a ligamentous mass, and a similar mass passes from the os triquetrum to the ulna.

The long head of the bicepo is missing, and we find no sulcus bicipitalis on the humerus for the reception of its tendon. The coraco-brachialis is poorly developed; the brachialis is very diminutive; the short head of the biceps unusually well developed. Anteriorily, at the bend of the elbow, a muscle passes from one condyle to the other, connected above with the brachialis, and below, with the extensor digitorum communis, and flexor digitorum subimis.

The supinator longus and brevis, extensor radialis longus and brevis, and the indicator are wanting. Only one tendon of the extensor digitorum communis passes through the ligamentum carpidorsale. The ulnaris externus and internus, and the flexor digitorum sublimis and profundus are present, but the sublimis is very poorly developed. In place of the long dorsal muscles of thumb, we find two muscles covered by the extensor digitorum communis, and the extensor digiti minimi, the upper one of which is inserted into the first phalanx of the index finger, and the lower one into the dorsal aponenrosis of the middle finger. Beneath these muscles, on the other side of the foramen, there are three other muscles, which have no counterpart in normal anatomy. The muscles of the thumb are wanting, and also the interosseus externus of the index finger.

As in other cases in which the radius is wanting, there were other deformities in other parts of the body. Notably, an incomplete development of the septum ventriculorum cardis; a slit in the left upper lip; an absence of the thumb of the right hand.—*St. Louis Courier of Medicine.*

